# The Emerging Role of Ultrasonic Nanotechnology for Diagnosing and Treatment of Diseases

**DOI:** 10.3389/fmed.2022.814986

**Published:** 2022-02-22

**Authors:** Xinying Liu, Weidong Ge

**Affiliations:** Department of Ultrasonography, Zhejiang Provincial People's Hospital, Affiliated People's Hospital, Medical College, Hangzhou, China

**Keywords:** diagnosis, nanoparticles, nanotechnology, treatment, ultrasonic

## Abstract

Nanotechnology has been commonly used in a variety of applications in recent years. Nanomedicine has also gotten a lot of attention in the medical and treatment fields. Ultrasonic technology is already being used in research as a powerful tool for manufacturing nonmaterial and in the decoration of catalyst supports for energy applications and material processing. For the development of nanoparticles and the decoration of catalytic assisted powders with nanoparticles, low or high-frequency Ultrasonic are used. The Ultrasonic is frequently used in joint venture with the nanotechnology from the past few years and bring tremendous success in various diseases diagnosing and treatment. Numerous kinds of nanoparticles are fabricated with desired capabilities and targeted toward different targets. This review first highlights the Ultrasonic Treatment and processing of Nanoparticles for Pharmaceuticals. Next, we explain various nanoparticles with ultrasonic technology for different diagnosing and treatment of various diseases. Finally, we explain the challenges face by current approaches for their translation in clinics.

## Introduction

The production of new compounds and applications has resulted from research into integrated approaches. During the past few decades, ultrasonic-assisted processes have intrigued the imagination of multidisciplinary scientists searching for more effective structures. A special paper on “Ultrasonic Nanotechnology of Ultrasonic Sonochemistry has given an insight into ultrasounds numerous applications. Ultrasound application has become the most imperative technologies to produce different natural amalgams because it is environmentally sustainable, hygienic, prolific, and resourceful. When hydrodynamic bubbles produced by high-octane waves of ultrasound collapse, micro-reactors with temperature and high pressure are created. Efficiency and product yield while speeding up chemical reactions in numerous processes. This question has been extensively discussed to improve the physicochemical properties of nanomaterials when generated by a sonochemical facilitated process. Siadatnasab at el. Reported that sonochemical reaction of methanolic Cu (II) diethyldithiocarbamate with phosphomolybdic acid formed a green precipitate of Cu3 nanohybrid, which was used for sonochemical degradation of Rhodamine B (RhB) (1). Another research group developed a scalable synthesis of tunable titanium nanotubes *via* sonoelectrochemical process (2). They used sonoelectrochemical process to synthesize TiO_2_ nanotube arrays on implantable Ti 6–4 structure were generated and tested using a sonoelectrochemical method as a drug delivery system for antibacterial applications (2).

A sonochemical-assisted process was utilized for synthesis and evaluation of nanostructured oil in water emulsions for targeted delivery of protein drugs (3). Protein extracted from a medicinal leech tissue was used to formulate nanoemulsion and sonochemical process was employed to form isotropic and kinetically stable nanoemulsion with least surfactant and optimal solubility and stability for drug delivery of protein drugs. (3). Allami et al. (4) discovered that waves of ultrasound produced biodiesel with lower viscidness, higher oxygen content, and thickness that improved fuel combustion. Ultrasound was used to clean a granulated surface by removing inadequate areas for dissolved substances (5). They utilized an ultrasound-assisted method for photocatalytic dye degradation and adsorbent regeneration to synthesize ZnO nanoparticles. The crystallization process is assisted by hydrodynamic bubbles using ultrasonic irradiation during the process. In this regard, Azarhoosh et al. (6) have used ultrasonic irradiation to complete the representation stage of a aluminophosphate-34 catalyst and evaluated the results of the parameters involved during this process for the adaptation of methanol with light olefins. Synthesis of ZnO nanoparticle was also performed using a sonochemical process, doped with numerous lanthanide cations (7). In a sono-photocatalytic membrane reactor, the produced photocatalysts were able to effectively oxidize the natural contaminant. Panahi et al. (8) used ultrasound waves for the manufacture and synthesis of benzimidazole of a porous zirconium/Aminophylline polymer co-ordination. Increased catalytic synthesis of benzimidazoles was achieved through the electron transfer mechanism below the control of ultrasound waves. Ultrasound waves are one of the most effective extraction methods currently available with safe profile to biological tissues. High-intensity sound waves can cause bioactive compounds to diffuse quickly into the solvent, resulting in a faster extraction time. Bayrami et al. used this technology to create biogenic ZnO nanoparticles for biomedical applications using leaf extracts from medicinally important plants like Nasturtium officinale L. and Vaccinium arctostaphylos L. (9, 10). These bio-synthesized photocatalysts have provided a gateway to several conservational refinement classifications due to improved photocatalytic properties.

ZnO nanoparticles waste was used as sonocatalysis for acetaminophen elimination. The UVC structure degraded acetaminophen to fewer toxic arbitrates (11). The sonocatalytic behavior of ZnTi nano-layered double hydroxide was greatly upgraded when a component of Zn2+ was replaced with Cu2+ (12). A primary reason for this change was the reduced band difference arising from the transition of charge to Cu(II) from Ti(IV) when bridged with oxygen atoms. Another study was carried through a radically mediated process to use znO-loaded nano-cellulose as a tetracycline sonocatalyst (13). The ultrasonically triggered nanocomposite contributed to nearly complete tetracycline dilapidation in combination with peroxymonosulfate. This review first highlights the ultrasonic treatment and processing of Nanoparticles for Pharmaceuticals. Next, we explain various nanoparticles with ultrasonic technology for different diagnosing and treatment of various diseases. Finally, we explain the challenges face by current approaches for their translation in clinics.

## Ultrasonic Treatment of Nanoparticles for Pharmaceuticals

Ultrasound is a pioneering technique used for synthesizing sonochemical, breaking down the agglomeration, blend, and activate particles. Ultrasound is a crucial technique for nanoscale materials, particularly in nanotechnology, to be synthesized and processed. Nano-sized particles are used in a wide variety of scientific and industrial fields as nanotechnology has gained such widespread scientific interest. This versatile and variable material's high potential has also been discovered by the pharmaceutical industry. As a result, nanoparticles are used in several different applications in the pharmaceutical industry. Drug distribution by nanoparticles is a validated process for supplying orally or injected active agents (14). When modern methods open up entirely novel avenues of medical care, nano-formulated medicines can be dosed and distributed even more efficiently. This high-potential technology aids in the delivery of medicines and temperature control to diseased cells. Side effects of drugs don't affect healthy cells because of direct drug delivery. Cancer therapy is one field where nano-formulated drugs have already shown promising results.

## Nanomaterials Processing

Nanomaterials have a diameter of under 100 nm and are known as particles. Their processing needs to be increased. Agglomerates should be dissolved nanoparticles process, and for bonding forces to shape. Ultrasonic hydrodynamic is a popular process for nanomaterial dispersion and decomposition. Nanomaterials come in a variety of forms and provide opportunities for medicinal research. The inner size of Carbon Nanotubes (CNTs) allows more drug molecules to be condensed and functionalized (14). DNA, active agents, and proteins targeting ligands, and other molecules may be carried into cells by CNTs. CNTs have established themselves as the archetypal nanomaterials, with nanoscience and nanotechnology being one of the most active fields.

**Figure 1:**
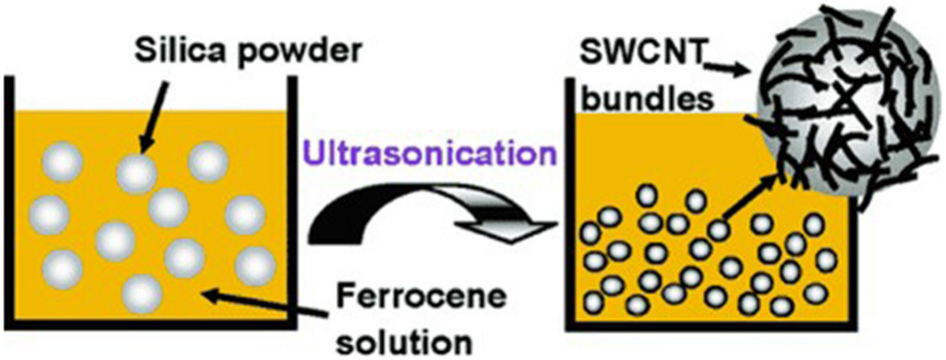
Sonochemical processing of SWCNTs. Shows that silica powder in a solution of the ferrocene-xylene combination was sonicated for 20 min under atmospheric pressure. Sonication produces highly pure SWCNTs on the surface of the silica powder.

SWCNT has a diameter of 1.0–1.4 nm and is much smaller. Cells can absorb nanoparticles and nanotubes (15). Functionalized Carbon Nanotubes (f-CNTs) improved solubility and allowed tumor targeting (Figure 1). A sonochemical process can be used to make high-purity single-walled carbon nanotubes (SWCNTs) (16).

The vaccines may be used for the delivery of functional Carbon Nanotubes (f-CNTs). The basic theory is to bind the antigen to the carbon nanotubes while retaining their shape, leading to a certain antibody reaction. Ceramic nanoparticles have a porous surface region that is suitable for the transmission of medicinal items.

**Figure 2:**
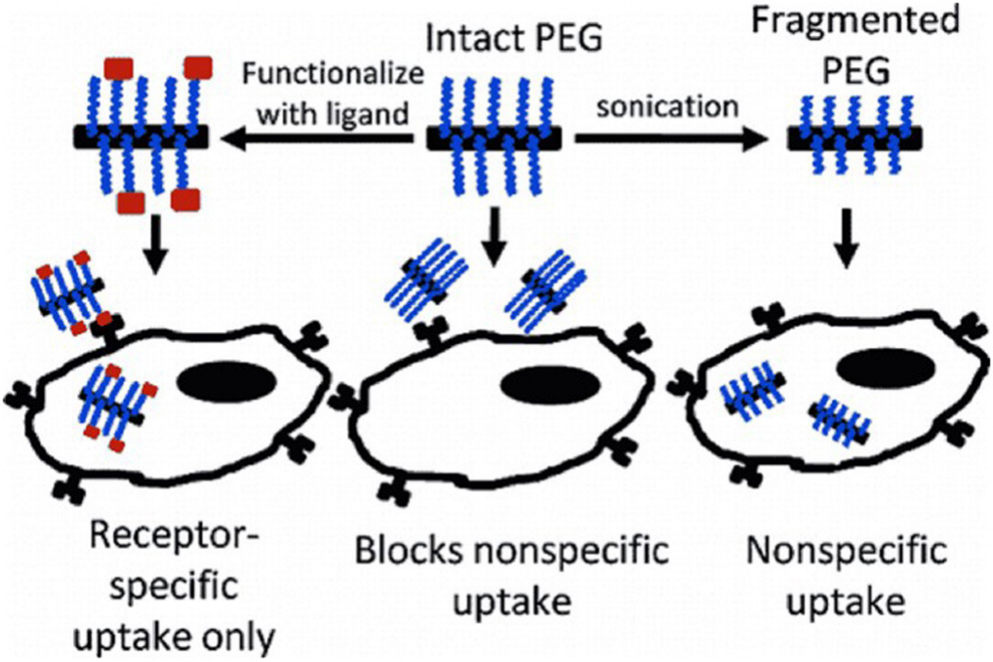
Ultrasonic dispersion of SWCNTs with PL-PEG.

Previous studies showed that ULTS of phospholipid-polyethylene glycol (PL-PEGs) fragments of (SWNTs) with the capacity to prevent cellular non-specific absorption. Unfragmented PL-PEG facilitates cellular absorption selective of targeted SWNTs in two different cell receptor groups. The integrity of PEG is crucial to help ligand-functional nanotubes' cellular absorption because fragmentation is a potential side effect of ultrasound for dispersing SWNTs (Figure 2) (17, 18).

## Conjugates of Drug Polymer

**Figure 3:**
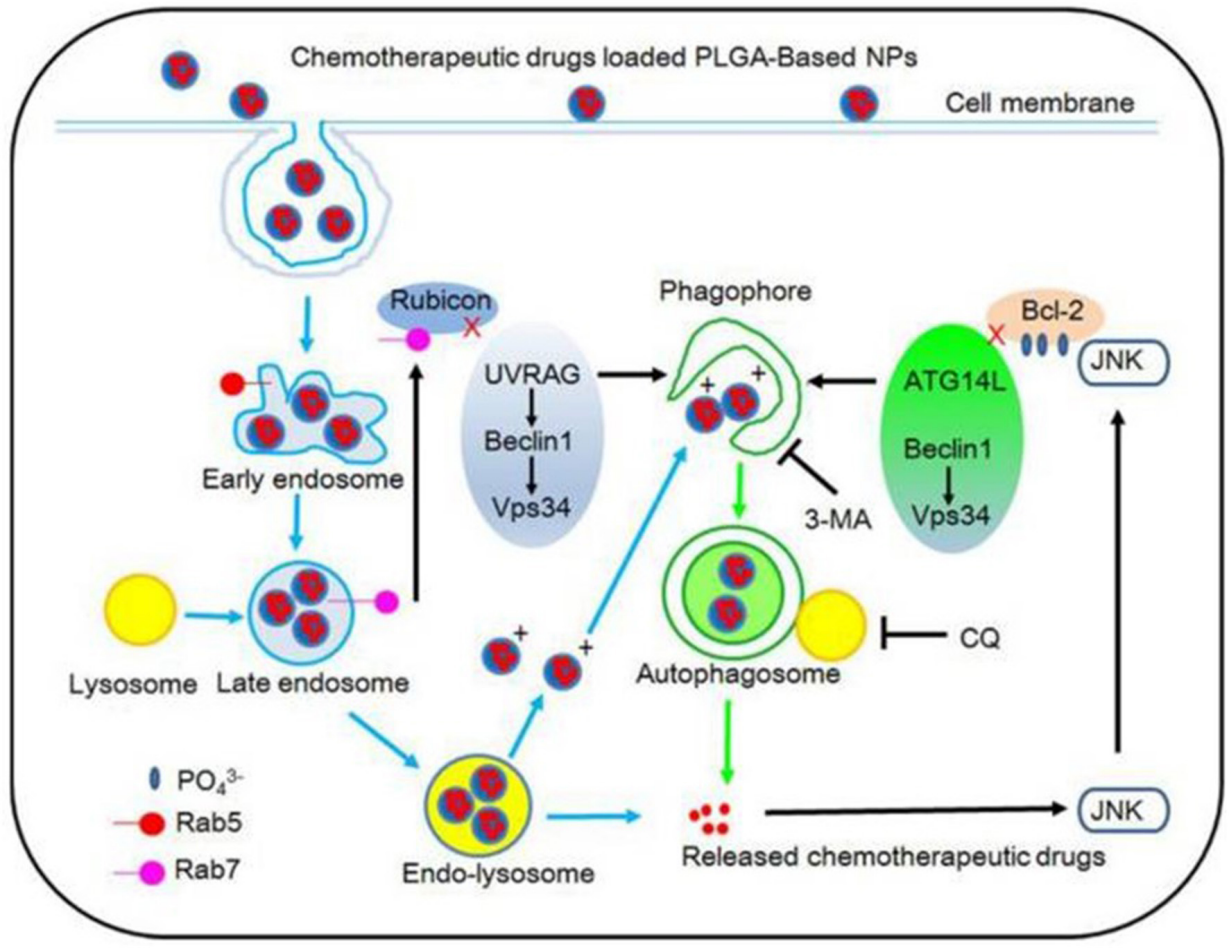
The degradation process of PLGA-based nanoparticles is depicted schematically. Reproduced with permission (35).

Drug-polymers conjugates are manufactured using diverse chemicals on the efficient groups of the pharmaceutical and polymer carrier. The two major types of conjugates are drug conjugates and protein conjugates with adequate polymers (19). The most active polymer of the theragnostic drug-polymer conjugates is N-(2-hydroxypropyl) methacrylamide (HPMA) (19, 20). Yuan et al. (21) recently created theragnostic copolymers based upon poly (HPMA) loaded with Cu-64 and RGD as a target ligand for the target of tumor ontogenesis (Figure 3). The drug-polymer conjugate of prostate cancer xerographers was tested by a positron emission tomography (PET) 3 h after intravenous injecting, the tumor Cu-64 radioactivity in rats. The pharmacokinetics of Cu-64 in tumor (21) increased by 1 time with the drug-polymer conjugate.

### Polymeric/Magnetic Nanoparticles

To produce polymer nanoparticles, monomer polymerization or polyp dispersion were used (22–26). Magnetite (27) nanoparticles of iron oxide are utilized as magnetic nanoparticles. Iron oxide nanoparticles have been extensively used due to their superparamagnetic properties and biocompatibility. The most practical methods for producing iron oxide nanoparticles are co-precipitation and thermal decomposition (27–31).

Due to their intrinsic theranostic properties, magnetic nanoparticles play a role as a hyperthermia agent with high transverse relaxation time (T2) for MRI and immunotherapeutic platforms for immunological diseases (28, 32–34). Recent evidence reported that autophagy pathway plays significant roles in targeting and degrading polymeric nanoparticles *via* auto-lysosomes (35). Polymeric nanoparticles are affected by cells *via* endocytosis and transferred *via* lysosomes that are an endosome pathway for degradation (Figure 3) (35, 36).

In breast cancer cells that overexpress folate receptors and fibroblast cells with a low number of pteroylmonoglutamic Acid receptors, the effect of quantum dots loaded nanoparticles on pteroylmonoglutamic Acid receptor was investigated in this study (37). Results showed that folate-decorated quantum dots loaded synthetic amphiphile nanoparticles were better than fibroblast cells. Synthetic amphiphile copolymers conjugated to targeting ligands may be a successful theragnostic approach for targeted diagnosis and treatment. PLA-TPGS nanoparticles were produced to syndicate their benefits and allow long-term, controlled imaging with a cancer cell. Biocompatibility and cellular absorption were improved because of this novel strategy by lowering their toxicity. The xenograft model was used to examine the biodistribution of the quantum dots and iron oxides loaded synthetic amphiphile nanoparticles among the different structures. *Ex vivo* fluorescent images showed a 51.5% increase in the kidney, 67.1% increase in fluorescent intensity in the liver, and 152.8% increase in the tumor. The blood-brain barrier surface adsorption of nanoparticles revealed that brain samples had more fluorescent signals than other organs.

**Figure 4:**
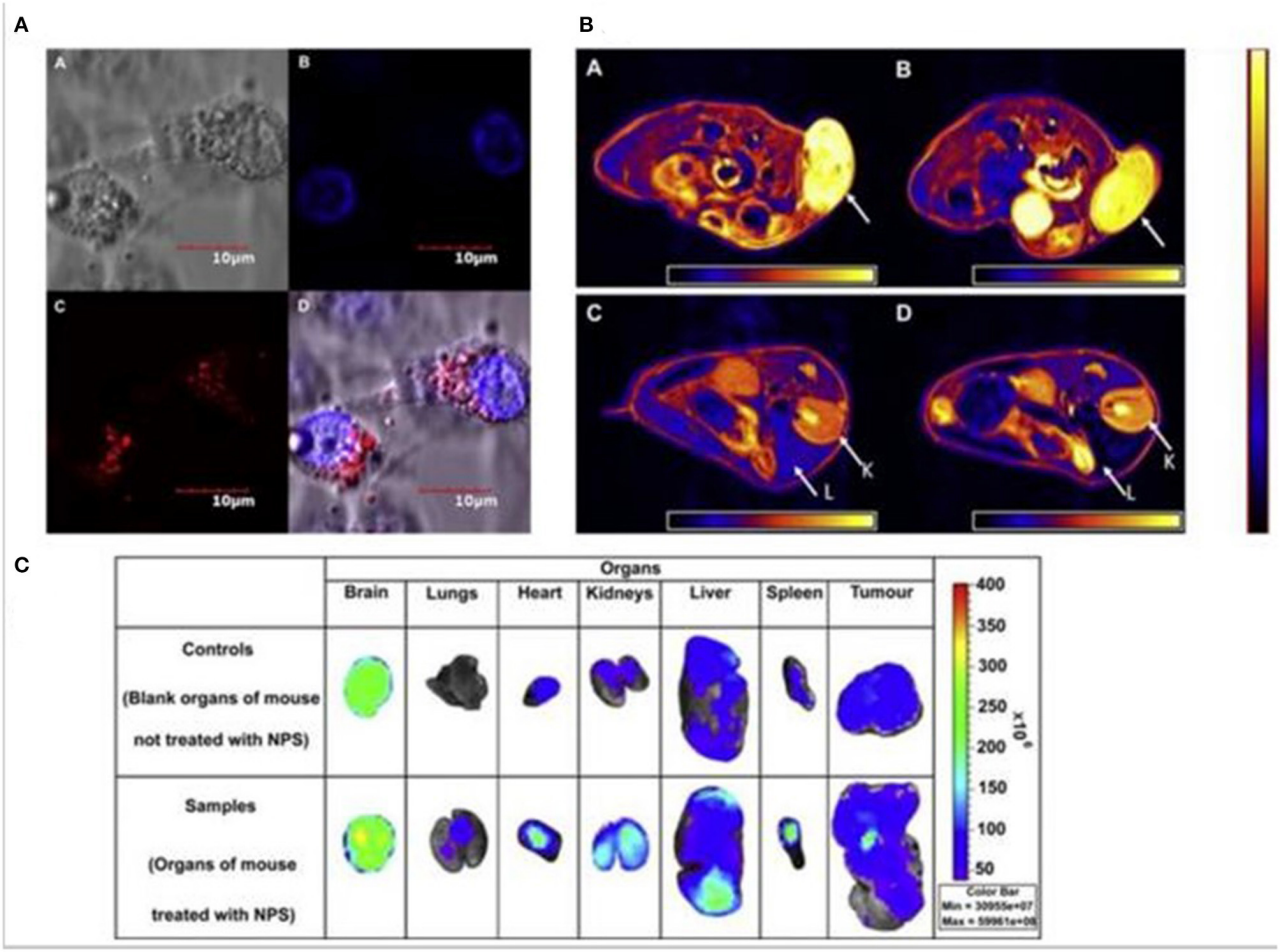
Multi-modal imaging nanoparticles **(A)** Confocal photographs of PLA-TPGS nanoparticles with quantum dots and iron oxides processed *in vitro* with MCF-7. **(B)** MCF 7-graphed tumor-bearing mouse portions of axial MRI photographs. **(C)** Pictures under fluorescent light of various organs. The arrow shows the intensity of the confocol microscopy.

**Figure 5:**
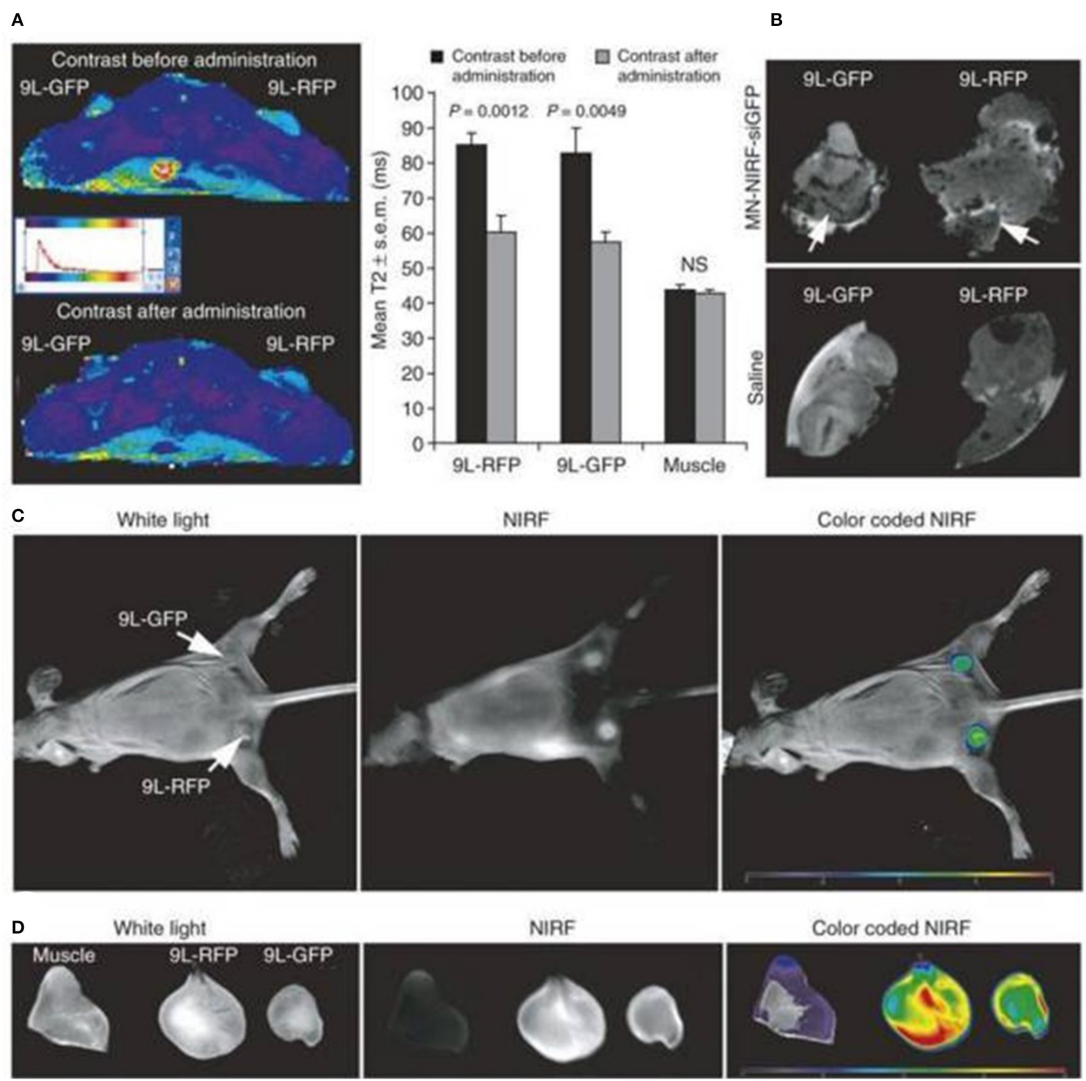
It reveals that *in vivo* MRI was conducted before and after 24 h the administration of nanoparticles (9L-GFP) (9L-RFP) on rats with tumors. **(A)** After the tumor was injected, T2 relaxation reduced dramatically. It should be noted that the T2 muscle tissue relaxation times have not improved. **(B)** *Ex vivo* excised tumor high-resolution RMIs (78 m isotropic). Differentiated signal loss (arrows) indicating the concentration of the probe is quickly recognized in tumors that resulted from muzzle-injected controls. **(C)** The optical *in vivo* NIR imagery of the same rat showed a tumor-based high-intensity NIR signal. This meant that the tissue had been penetrated by the nanoparticle sample. **(D)** *Ex vivo* NIR optic imaging showed a large increase in fluorescence in the polyps substantially more than muscle tissue (*P* = 0.0058). Reproduced with permission from Medarova et al. (38) and Muthu and Singh (39).

Inadequate biodistribution of the quantum dots and iron oxides loaded synthetic amphiphile nanoparticles through the selective semipermeable border of endothelial cells (Figure 4). The advantages of multimodal imaging system, which results in a probe that is extremely sensitive and has deep infiltration for up to 6 h, confirming the diagnosis made by every individual's imaging. It was also proposed that using this multimodal approach to encapsulate therapeutics and conjugate ligands resulting in the progress of advanced multimodal theragnostic nanomedicine. Medarova et al. (38) developed the use of high resolution *in vivo* optical MRI, NIR and iron oxides for simultaneous imagery and siRNA distribution in tumors. N-succinimidyl-3 propionate was utilized to bind siRNA finished dextran particles to bridge the surface area of iron oxide nanoparticles and later the NIR dye Cy5.5 was also coupled to the surface area. The siRNA dissemination and its silencing capabilities were monitored with MRI and NIR optical imaging for 48 h using dextran coated iron oxide nanoparticles (Figure 5) (38).

### Solid Lipid Nanoparticles

Nanoparticles of stable lipid are a secure and efficacious substitute for intravascular supply (39). They have a strong hydrophobic heart with medicine inside. Nanomedicine made from biocompatible lipid substances solid at room temperature is Stable lipid nanoparticles. The warm and cold amalgamations are two significant approaches of preparation. Strong lipid nanoparticles enter the blood cell easily because of their compact size and lipotropic surface. The tightened endothelial cell blood-brain barrier is crossed by strong lipid nanoparticles with a range of <100 nm. The high efficiency of medication loads keeps the medication steady in the strong lipid matrix and makes the controlled release (40–42). As for the theranostic platforms for selective co-delivery of diagnostic and therapeutic agents, solid lipid nanoparticles like other nanomedicines are utilized (42, 43). Lymphatic delivery of nanoparticles of solid lipid has been developed as a technique to improve the transfer of beneficial agents into the lymphatic environment, which leads to improved oral bioavailability (44). Bae et al. (45) reported the applications of paclitaxel and siRNA loaded in solid lipid nanoparticles as theranostic anti-cancer agents with beneficial outcomes. The solid lipid nanoparticles were electrically complexed with the exterior surface of solid lipids and were generated with a stable core nanostructure like quantum dots and paclitaxel in the lipid shell, resembling low-density lipoproteins (LDL).

### Dendrimers

Dendrimers are a form of synthetic nanomedicine made up of a spherical polymer with a lot of branches. Nanotheranostic systems usually use dendrimers that are 10–100 nm in size (46). Dendrimers can be made in two ways: beginning from the central core and moving outwards (divergent synthesis) or starting from the periphery and working inwards (top–down synthesis) (convergent synthesis). They are made by adding branching units to an amine center over and over again (ethylenediamine or ammonia). Dendrimers are repeatedly rounded by a branching sequence that leads to an early, perfect 3D geometric pattern. Dendrimers were harmful as cell membranes were damaged by their positive surface load. Dendrimers encapsulated drugs are prone to escape fast before hitting the target location in some cases (47, 48). The polymerization degrees are regulated by the synthesis of dendrimers of various sizes, molecular weights, and chemical compositions (49, 50). Theranostic dendrimers have a circular structure that holds both therapeutic and diagnostic agents with several cavities and divisions. The 5th generation of dendrimers with higher hydrophobic value is typically preferred in dendrimers (47–50). Poly (amidoamine) dendrimers generation 5 is coated with a replicant adenovirus serotype 5 carrying the sodium-iodide symporter and tested for transudative efficacy in a liver cancer xenograft model using an I-123 scan. *In vitro*, adenovirus serotype showed partial resistance to dendrimer-coating neutralization antibody and increased transduction efficiency in coxsackie adenovirus receptor-negative cells.

The main limitations impeding the clinical applications of adenovirus-mediated gene therapy are excess expression of coxsackie-adenovirus receptor (CAR), excess presence of neutralizing antibodies, and adenovirus sequestration by the liver (51, 52). Recent studies have exhibited the capacities of dendrimers to overcome these limitations through coating of the adenovirus to build adenoviral vectors (52–56). Different studies have reported successful use of this approach in cancer therapy. They used synthetic dendrimers to coat sodium iodide symporter (NIS) as a theranostic gene to develop adenoviral vectors for combination of systemic oncolytic virotherapy and NIS-mediated radiotherapy (54, 55, 57, 58). Taratula et al. (59) designed a new dendrimer-based theranostic system for phthalocyanines (Pc) delivery to tumors for tumor-targeted delivery of phthalocyanines Adding a hydrophobic linker to the Pc molecule during the preparation stage makes physical encapsulation of the hydrophobic compound into a generation 4 polypropylene imine (PPIG4) dendrimer much easier. To boost biocompatibility and tumor-targeted delivery with up to 24 h of photodynamic therapy, PEG and LHRH peptides were applied to the surface of the Pc-PPIG4 complexes. The LHRH-targeted theranostic dendrimer is capable of successful internalization into cancer cells as well as tumor aggregation, according to *in vitro* and *in vivo* imaging studies (59–67).

### Liposomes

**Figure 6:**
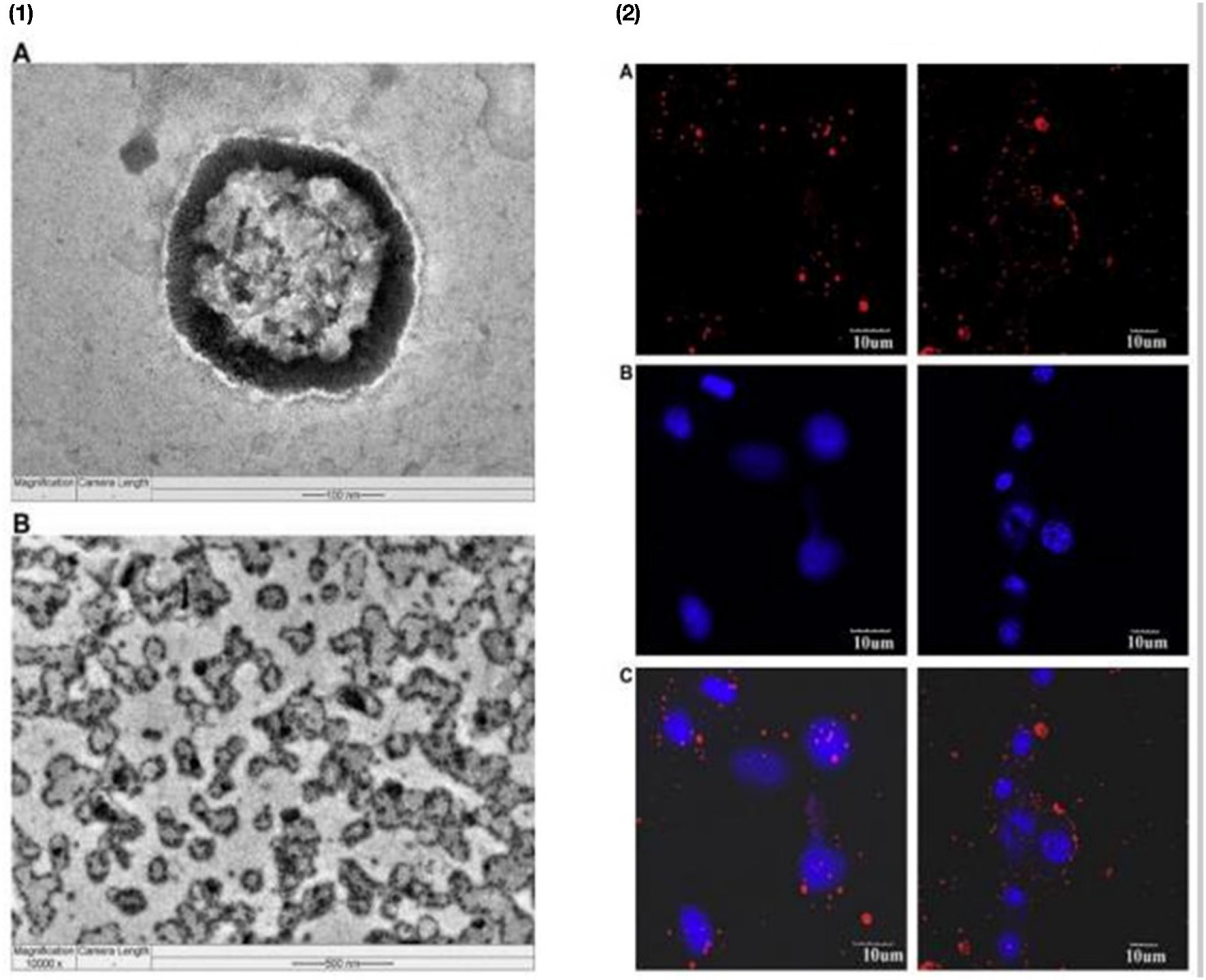
TPGS liposomes were used to transport theranostic drugs *in vitro*. **(1) (A)** A single quantum dots-loaded TPGS coated liposome in a 100 nm scale, and **(B)** multiple quantum dots-loaded TPGS coated liposomes after storage in a 500 nm scale. **(2) (A,B)** Confocal laser scanning microscopy images of MCF-7 cells treated with non-targeted TPGS-based multi-functional liposomes (left column) and targeted TPGS-based multi-functional liposomes (right column) for 2 h (right column). Quantum dots showing red fluorescence from cytoplasmic liposomes, **(B)** channels displaying blue fluorescence from dye-stained nuclei, and **(C)** quantum dots displaying blue fluorescence from dye-stained nuclei, and **(C)** Quantum dots and blue dye merged channels.

Liposomes are composed of amphiphilic phospholipids and cholesterol (68). Liposomes are spherical particles with a diameter ranging from 400 to <400 nanometers (62, 69). Liposomes are effective vectors for drug/diagnostic delivery due to their size, hydrophobic and hydrophilic nature, biodegradability, biocompatibility, and immunogenicity. Mechanical dispersion, solvent dispersion, and detergent removal are the three most popular liposome preparation processes. Liposomes have several drawbacks, including poor drug loading efficiency, batch-to-batch manufacturing volatility, and poor stability (70–72). Beneficial agents may be encapsulated in the middle or integrated into the lipophilic bilayer shell while nanosized diagnostic agents such as iron oxide nanoparticles, quantum dots, and gold nanoparticles may be incorporated in the lipophilic bilayer shell (71, 73–77). Advanced theranostic liposomes are conjugated with molecular biomarkers for a targeted outcome. To resolve immune system opsonization and fast elimination from circulation, stealth liposomes, or PEG-coated liposomes, were established with stability and a longer half-life in blood (74, 75, 78). PEGylated liposomes, standard nude liposomes (without TPGS coating), and TPGS coated liposomes were tested *in vitro* on cell lines to see whether they could guard against brain tumors and were found to be more effective than those coated with PEGylated liposomes (79). Muthu et al. (80) rendered TPGS-coated theranostic liposomes with and without docetaxel and quantum dots targeting moieties. Targeted theranostic liposomes exhibited higher cellular absorption and cytotoxicity than non-targeted liposomes (Figure 6).

### Micelles

Micelles are hydrophobic, hydrophilic structures with a self-assembling hydrophilic core for parenteral management of products that are badly water-soluble (81–83). The main synthesis method for micelles in nanoscale is dispersion of surfactants in water which then generates a two-component micelles with a hydrophilic shell and a hydrophobic core (84–86). Polymeric micelles are self-assembled and aggregated nanoscale assemblies with diameter of ≤100 nm consisting of amphiphilic copolymers enclosed with an aqueous phase. The hydrophobic and neutral parts of copolymers are bound together due to an attractive force between them, the process which facilitates the micellization. The micellization process depends on the micelle concentration in a manner that when the micelle concentration reaches a threshold level called critical micelle concentration (CMC), the micellization process starts (87, 88). The stability of the micelles is determined by the strong cohesive force between the drug and the center of the polymer and the crosslinking of the core or shell. The mechanism of direct dissolution and the organic solvent system is usually used to produce them (89). The hydrophobic core of micelles, which can be given intravenously in and the outer hydrophilic layer using a targeting agent can be filled with diagnostic agents (90–93). Theranostic micelles with <50 nm of renal escape diameter improve the permeability of the endothelial cell and theranostic reticulate system provide solid tumors (94, 95). The paclitaxel-charged micellar formulations of Genexol-PMTM are approved to be an effective standard for the provision of cancer drugs (96–99).

### Gold Nanoparticles

Gold nanoparticles with gold nuclei are another versatile medium with desirable values for theranostic systems (100–103). They are made of 1.5% to 10 nm core sizes, offering a powerful drug and ligand conjugation with an expanded surface area 114. Hydrogen tetrachlorocuprate is a common method of preparing gold nanoparticles in the chemical treatment process. Gold nanoparticles can be combined as advanced theranostics with drugs and ligands to precisely identify the target receptor for successfully targeting (104, 105). Therapeutic loading is carried by non-covalent interaction or covalent chemical conjugation. The inherent characteristics of gold nanoparticles are diagnosis characteristics (104–107). Heo et al. (108) defined surface-functionalized Gold nanoparticles of PEG as the theranostic platform associated with beta-cyclodextrin (beta-CD). The inclusion complex Paclitaxel and beta-CD is bound to gold nanoparticles. Gold nanoparticles are more related to cancer cells such as HELA, A549, and MG63 than NIH3T3.

### Carbon Nanomaterials

Carbon nanomaterials or nano carbons were analyzed for theranostic purposes because of their peculiar chemical and physical qualities (109). Carbon nanomaterials such as carbon nanotubes (CNT), zero-dimensional (0D), sp2-carbon nanomaterials (sp2-carbon nanomaterials), 2D graphene, and carbon point with the size of nano-clusters smaller than 10 nm (109, 110). Due to its large area, its ability of diagnostic agents, and its aptitude for surface modifications (111–113), CNTs were considered suitable for theranostic applications. CNTs have a cylindrical form due to their various graphene sheet layers. Two kinds of carbon nanotubes are SWCNTs or MWCNNTs. CNTs are common methods for ball-milling, laser therapy, and chemical vapor deposition method (111, 114–118). In recent years, multifunctional CNT-based systems for theranostic applications have resulted from many synthetic methods for CNT functionalization. The theranostic applications for photoluminescent *in vivo* tumor imaging in the 1.0–1.4 m injected intravenously injected SWCNTs and NIR absorbers and heaters at 808 nm for lower doses for photothermal removal were seen by Robinson et al. (119).

Theranostic MWCNTs were first developed by Das et al. (120), *via* mixing acid oxidized MWCNTs with four distinct functional drive elements according to cellular uptake studies.

## Ultrasonic Nanoparticles Translation Challenges in Clinics

### Biological Challenges

Theranostic nanomedicine has plenty of research into disease diagnosis and treatment to improve human health. Nano theranostics remains a new paradigm for disease detection and care in hospitals. One of the toughest things to bring theranostic nanomedicine to clinics is nano-bio engagement. In interactions with biological materials, nanomedicine can cause problems such as inflammation and other diseases due to its potential toxicity depending on the potential and solubility of different parameters (121–123). A pseudo-allergy linked to complementary activation is an immediate adverse immune response from several nanoplatforms (124, 125). Study about pathophysiology and disease heterogeneity is imperative to the physicochemical characteristics of nanomedicines. Besides, as theranostic nanomedicine is individually distinct, it would be very difficult to have therapeutic clearance for a single-size solution (126). Nanoparticles with high therapeutic properties can not necessarily be good screening instruments, Consequently, the safety profile of human nano theranostics remains a major concern, which needs long-term surveillance of all early and advanced stages of clinical trials (127).

### Challenges of Commercialization

The challenge in designing a synthesis technique is also a key concern with the clinical translation of theranostic nanomedicines. Poor reproductivity and low efficient large-scale synthesis, and variable physico-chemical characteristics are common challenges toward clinical applications of nanoparticles. Nanoplatforms with complex manufacturing methods are barely incorporated into clinical practice due to disadvantages caused by drug companies (126, 127). Another big issue that needs to be addressed is the broad gap between the research community and regulatory authorities. Many government regulations are used to restrict the commercialization of nanomedicine based on regulatory considerations relevant to quality and manufacturing standards. There is an important effect on a prompt, effective translation of theranostics into the industry (128, 129). However, these criteria may not be satisfactory and need to be updated to validate the performance effectiveness of other human-using nanotheranostics.

### Clinical Considerations and Perspectives

The first nanocarrier for drug delivery, approved by the US FDA was Doxil (PEGylated liposomal doxorubicin) and designed for delivery of chemotherapeutic agent doxorubicin (130). The nanoparticles facilitated Doxil, exhibit several advantages over free doxorubicin including selectivity, specificity, and reduced cardiotoxicity (131). The successful clinical outcomes of Doxil in cancer therapy has led to the development of many other nanoscale carriers. Although nanotechnology-assisted theranostic systems are promising inventions in medicine, concerns on the safety of these nanosystems due to unknown characteristics of nanoscale materials have impeded the clinical applications of these systems. To address the concerns on the safety and understand the safety profile conducting animal studies, laboratory experiments and clinical trials is necessary. Stability of nanosystems is other important aspect of nanosystems for successful translation into clinical practice. Exposure to human subjects is inevitable to address these issues. Experiments in human subjects are complementary to *in vitro* and *in vivo* animal studies (132, 133). Failure in human studies can be too expensive and sometimes irrecoverable. Therefore, it is necessary to adopt a novel standardized nano-safety platform to develop reliable systems and avoid potential candidates with adverse outcomes (134).

Considering these risks and the unique characteristics of nanosystems, FDA necessitates conducting preclinical studies involving animals, human cells, or tissues prior to any study on human subjects as clinical trials for any nanoscale medications. Following the evaluation of FDA on the outcomes of the preclinical studies, they allow for Phase I clinical trials which is a dose-response trial with a small group of subjects (sample size: 25–100) to determine the maximum tolerable dose for the target product. These studies are followed by Phase II trials with greater sample size including 100–500 subjects, which evaluate the safety and efficacy of the developed nanomedicine. FDA allows conducting Phase III trials (sample size of 500–3,000 subjects) for those drugs that passed the Phase II clinical trials, and it is determined whether the application will be approved or not based on the findings of the Phase II (133).

To overcome the biological challenges to nanotheranostics, a great deal of research needs to be done about how the interaction between patient biology and nanomedicine is to be understood. In preclinical trials, animal models can be used to assess the appropriateness of theranostic nanomedicine in the treatment and imagery of patient populations of human beings (135). To evaluate future patient risk, nanotoxicology profiles need to be adopted and followed during the early stages of clinical development (124–129, 135–137). Recent advances in nanotheranostics have utilized improved permeability and retention as well as other characteristics of nanoparticles such as surface functionalization, selectivity and sensitivity, and biodegradability.

This would have enormous potential for theranostic applications in developing bio-mimetic nanoparticles, which exploit the normal functioning of the source. The effect of theranostics can be further improved by the use of smart stimuli-based nanoparticles to release therapeutic loads on the site. This method of provision and real-time analysis would help the clinician to adjust a care plan for heterogeneous and adaptive diseases. A mindful awareness needs to spread for the technical problems facing the industry in marketing systems. Good collaboration is required between laboratory and pharmaceutical groups. For large-scale theranostic nanoparticles synthesis and improvements on good manufacturing, practice must be made. Process optimization applications like Aspen is useful in an industrial setting to define key parameters to maximize performance in the early stages of manufacture and cope with batch-to-batch variations. This could be in a supervised and efficient way (138).

Production success depends on the readiness of the employees for product specifications and barriers. Theranostic nanomedicines can affect human health, but by incorporating the above lessons in the early stages of manufacturing, manufacturers can produce efficient products.

## Conclusion

While ultrasound nanomedicine has dramatically progressed and is continuing to make substantial progress, the field must evolve before human medicine can transform. Nanotheranostics are supplementary to nanomedicine that could be used in medical centers to monitor disease. Nanotheranostics is promising for a deeper understanding of the therapeutic and diagnostic interwoven substances that are necessary to maximize their clinical application potential. Besides, both commercialization and regulatory stages need to be followed by the most promising approaches for bringing ultrasonic nanomedicine from research laboratory studies into clinics.

## Author Contributions

XL and WG downloaded the references and processed the graphs in the manuscript. XL wrote the first version and WG finalized the manuscript. WG conceived and coordinated the study and critically evaluated the data. All authors read and approved the final manuscript.

## Funding

The research was supported by Medical and Health Research Project of Zhejiang Province (No. 2019KY303).

## Conflict of Interest

The authors declare that the research was conducted in the absence of any commercial or financial relationships that could be construed as a potential conflict of interest.

## Publisher's Note

All claims expressed in this article are solely those of the authors and do not necessarily represent those of their affiliated organizations, or those of the publisher, the editors and the reviewers. Any product that may be evaluated in this article, or claim that may be made by its manufacturer, is not guaranteed or endorsed by the publisher.
